# Effect modification of late surfactant treatment by patent ductus arteriosus status in ventilated preterm infants: a secondary analysis of a randomized clinical trial

**DOI:** 10.1038/s41372-026-02705-x

**Published:** 2026-04-20

**Authors:** Patrick J. Peebles, Jens C. Eickhoff, Timothy G. Elgin, Anudeepa Sharma, Nicolas A. Bamat, Jonathan M. Klein, Patrick J. McNamara, Roberta L. Keller, Dinushan C. Kaluarachchi

**Affiliations:** 1 Division of Neonatology, Department of Pediatrics, University of Wisconsin School of Medicine and Public Health, Madison, WI USA; 2 Department of Pediatrics, University of Wisconsin School of Medicine and Public Health, Madison, WI USA; 3 Division of Neonatology, Department of Pediatrics, Children’s Hospital of Philadelphia, University of Pennsylvania, Philadelphia, PA USA; 4 Division of Neonatology, Department of Pediatrics, University of Iowa Hospitals and Clinics, Iowa City, IA USA; 5 Division of Neonatology, Department of Pediatrics, University of California San Francisco, San Francisco, CA USA

**Keywords:** Respiratory tract diseases, Respiratory signs and symptoms, Risk factors

## Abstract

**Objective:**

To evaluate whether the effect of late surfactant is modified by patent ductus arteriosus (PDA) status with respect to survival without bronchopulmonary dysplasia (BPD) in ventilated infants born <28 weeks’ gestation.

**Methods:**

Secondary analysis of the Trial of Late Surfactant (TOLSURF). A formal test of interaction between PDA presence and late surfactant, with respect to survival without BPD at 36 and 40 weeks’ postmenstrual age (PMA), was performed. PDA presence was defined by PDA treatment, either medical or a ligation (definition A), or PDA ligation (definition B), during or after late surfactant administration.

**Results:**

All 511 infants were included. No interaction was detected between a PDA and late surfactant for survival without BPD at 36 weeks’ PMA (definition A: *p* = 0.41; definition B: *p* = 0.33), or at 40 weeks’ PMA (definition A: *p* = 0.84; definition B: *p* = 0.42).

**Conclusion:**

PDA presence did not modify the effect of late surfactant for survival without BPD.

## Background

Bronchopulmonary dysplasia (BPD) is a significant source of morbidity among extremely preterm infants. The rate of bronchopulmonary dysplasia (BPD) among extremely preterm infants remains high over the past several decades despite an improvement in mortality [1–3]. The use of late surfactant, administered in the second postnatal week, has been studied as a potential therapy to reduce BPD in this high-risk population in several clinical trials [4–8]. These trials followed a single center observational study showing improvements in respiratory severity scores (RSS) within 48 h among 18 of 25 (72%) extremely low birth weight infants who received late surfactant [9]. Although late surfactant did not improve survival without BPD, infants that received late surfactant had improved post-discharge respiratory outcomes [10].

The largest of the clinical trials was the Trial of Late Surfactant (TOLSURF) that enrolled 511 infants born <28 weeks’ gestation [6]. All infants in TOLSURF received co-administration of inhaled nitric oxide (iNO), which may increase pulmonary blood flow in patients with a patent ductus arteriosus (PDA). A recent observational study in 35 extremely preterm infants found an association between the presence of a PDA and a negative response to late surfactant, measured by a ≥ 15% increase in RSS over the next 48 h [11]. A negative response to late surfactant in the setting of a PDA can be physiologically explained by surfactant improving lung compliance and lowering pulmonary vascular resistance. Subsequently, the lower pulmonary vascular resistance leads to increased pulmonary blood flow, pulmonary edema, and worsening respiratory function. Given these findings, the presence of a PDA may impact the effect of late surfactant by worsening lung compliance, which may be further aggravated by increased pulmonary blood flow in the setting of iNO administration. Therefore, it is possible that the overall lack of improvement in survival without BPD observed in these trials was related to worse respiratory outcomes among the subset of patients with a PDA.

## Objective

To determine whether the effect of late surfactant is modified by PDA status with respect to the primary outcomes of survival without BPD at 36 weeks’ postmenstrual age (PMA) and survival without BPD at 40 weeks’ PMA, and several pre-specified secondary outcomes, including components of the primary outcomes and later respiratory outcomes.

## Methods

We performed a post hoc secondary analysis using data from TOLSURF. We analyzed all covariates and outcomes as they were originally reported.

### Study participants

The 511 patients enrolled in TOLSURF were born <28 weeks’ gestation and received mechanical ventilation between 7 and 14 postnatal days at 25 different medical centers in the United States between January 2010 and September 2013. All patients received iNO for a 25-day course per the study protocol and were randomized to either surfactant (calfactant) or a sham procedure up to a maximum of 5 doses if they remained intubated.

### PDA exposure

In TOLSURF, echocardiography data regarding PDA presence were not collected; however, data were collected regarding PDA medical treatment and PDA ligations, along with the timing of the treatment with respect to the study intervention. All treatment decisions regarding PDAs were made at the discretion of the clinical teams. We defined PDA presence using available PDA treatment data and evaluated two different definitions. PDA definition “A” defined the presence of a PDA as either medical treatment or a ligation, during or after the late surfactant dosing period. PDA definition “B” classified PDA presence as a PDA ligation during or after the late surfactant dosing period. For each definition, patients who did not meet criteria for PDA presence were classified as “No PDA.” Any patient who received a PDA ligation prior to the late surfactant dosing period was classified as “No PDA” by either definition.

### Outcome measures

We evaluated two primary outcomes as defined in TOLSURF, which were adjudicated in the parent study: (1) survival without BPD at 36 weeks’ PMA, and (2) survival without BPD at 40 weeks’ PMA. Secondary outcomes included persistent pulmonary morbidity by one-year corrected age, no pulmonary morbidity by one-year corrected age, BPD at 36 weeks’ PMA, BPD at 40 weeks’ PMA, death before 36 weeks’ PMA, death before 40 weeks’ PMA, and additional outcomes we derived from the TOLSURF dataset: the composite of grade 2 or 3 BPD or death before 36 weeks’ PMA [12], the composite of grade 3 BPD or death before 36 weeks’ PMA, home respiratory support after discharge, mechanical ventilation ≥37 days (median for the cohort), and total NICU days ≥106 (median for the cohort).

In the TOLSURF study, pulmonary morbidity was assessed by caregiver surveys focusing on respiratory medications, hospitalizations, and home respiratory support at 3, 6, 9, and 12-months’ corrected age. The definitions of BPD, persistent pulmonary morbidity, and no pulmonary morbidity are shown in Table 1.

**Table 1:** TOLSURF respiratory outcomes definitions.

BPD at 36 or 40 weeks postmenstrual age	Any of the following • Any level of supplemental oxygen, or with an effective FiO2 > 30% by nasal cannula. • Mechanical ventilation, NCPAP or >4 L of nasal cannula • Failed oxygen/flow reduction challenge test (unable to maintain oxygen saturations greater than or equal to 90% in room air)
Persistent pulmonary morbidity	Infants with morbidity in any respiratory domain (medications, hospitalization, home support) on at least 3 caregiver surveys through 12 months’ corrected age.
No pulmonary morbidity	Infants with no respiratory medications, hospitalizations, or home support on any survey through 12 months’ corrected age.

### Statistical analysis

Demographic and baseline clinical characteristics were summarized using medians and interquartile ranges for continuous variables, and frequencies and percentages for categorical variables. Comparisons between PDA status groups were performed using the nonparametric Wilcoxon Rank Sum test for continuous variables and the chi-square test or Fisher’s exact test for categorical variables, as appropriate. Multivariate logistic regression analyses were conducted to evaluate the interaction effects between treatment (late surfactant vs. control) and presence of PDA by both definitions on the primary binary clinical outcomes, including survival without BPD at 36 and 40 weeks PMA. The models were adjusted by gestational age at birth, birthweight, sex, multiple gestation, maternal age, respiratory severity score (RSS; calculated as FiO2 multiplied by mean airway pressure in centimeters of water) at enrollment. Similarly, multivariate logistic regression analyses were performed to evaluate the interaction between treatment group and PDA status on secondary binary clinical outcomes. No adjustments were made for multiple comparisons. The analyses were conducted using available data; missing data were treated as missing at random and were excluded from the analyses. All reported *p* values are two-sided, and a *p* value < 0.05 was considered statistically significant. Statistical analyses were conducted using SAS software (version 9.4; SAS Institute, Cary, NC).

### Ethics approval and consent to participate

A parent for each infant provided written informed consent, and the original study research protocol was approved by the Institutional Review Boards (IRB) at all of the participating institutions. This secondary analysis of TOLSURF was reviewed by the IRB at the University of Wisconsin who determined that the current analysis is not research involving human subjects as defined by DHHS and FDA regulations. This secondary analysis was conducted in adherence to all applicable guidelines and regulations.

## Results

All 511 infants from TOLSURF were included in the analysis; of these, 252 received late surfactant, and 259 were in the control arm. 184 infants met criteria for PDA definition A, and 106 met criteria for PDA definition B (ligation). The birth gestational age was lower for infants with a PDA compared to those without a PDA using either PDA definition A (PDA: 25.0 weeks, No PDA: 25.3 weeks, *p* = 0.01) or definition B (PDA ligation: 24.9 weeks, No PDA ligation: 25.3 weeks, *p* = 0.01). There were no significant differences in the other baseline characteristics (Table 2).

**Table 2:** Baseline characteristics, stratified by PDA status.

	PDA Definition A			PDA Definition B		
	PDA *N* = 184 Median [IQR] or (%)	No PDA *N* = 327 Median [IQR] or (%)	*P* value	PDA Ligation *N* = 106 Median [IQR] or (%)	No PDA Ligation *N* = 405 Median [IQR] or (%)	*P* value
Birth gestational Age (weeks)	25.0 [24.1–25.7]	25.3 [24.3–26.3]	0.01	24.9 [24.0–25.7]	25.3 [24.3–26.1]	0.01
Birthweight (g)	670 [588–779]	705 [590–812]	0.14	670 [580–770]	700 [590–800]	0.26
Male sex	95 (51.6)	186 (56.9)	0.25	57 (53.8)	224 (55.3)	0.78
Maternal Race/ethnicity			0.58			0.25
Black	65 (35.3)			38 (35.9)	150 (37.0)	
Hispanic	25 (13.6)	123 (37.6)		16 (15.1)	40 (9.9)	
White	84 (45.7)	31 (9.5)		45 (42.5)	199 (49.1)	
Other	8 (4.4)	160 (48.9)		5 (4.7)	14 (3.5)	
More than 1 race	2 (1.1)	11 (3.4)		2 (1.9)	2 (0.5)	
Antenatal Corticosteroids	159 (86.4)	281 (85.9)	0.88	93 (87.7)	347 (85.7)	0.59
Respiratory Severity Score at enrollment	3.0 [2.2–4.7]	3.2 [2.3–4.5]	0.34	3.2 [2.3–4.8]	3.1 [2.2–4.4]	0.30
Randomized to Late Surfactant	89 (48.4)	163 (49.9)	0.75	55 (51.9)	197 (48.6)	0.55

Among infants with a PDA per definition A, the incidence of survival without BPD at 36 weeks’ PMA was 28.1% in the late surfactant group compared to 23.4% in the control group (adjusted odds ratio 1.09, 95% confidence interval 0.66–1.79). Among infants without a PDA (definition A), the incidence of survival without BPD at 36 weeks’ PMA was 33.3% in the late surfactant group compared to 36.6% in the control group (adjusted odds ratio 0.97, 95% confidence interval 0.75–1.26). No interaction was detected between the presence of a PDA by definition A and late surfactant (*p* = 0.41) (Table 3).

**Table 3:** Interaction between PDA (definition A) or PDA Ligation (definition B) and the study primary outcomes.

Outcome	Subgroups	Late surfactant *N* = 252	Control *N* = 259	Adjusted odds ratio* (95% CI)	*P* value for interaction
Survival without BPD at 36 weeks’ PMA	PDA	25/89 (28.1%)	22/94 (23.4%)	1.09 (0.66–1.79)	0.41
	No PDA	54/162 (33.3%)	60/164 (36.6%)	0.97 (0.75–1.26)	
Survival without BPD at 36 weeks’ PMA	PDA ligation	15/55 (27.3%)	12/51 (23.5%)	0.86 (0.45–1.66)	0.84
	No PDA ligation	64/196 (32.7%)	70/207 (33.8%)	1.05 (0.81–1.36)	
Survival without BPD at 40 weeks’ PMA	PDA	51/89 (57.3%)	43/93 (46.2%)	1.09 (0.84–1.41)	0.33
	No PDA	97/162 (59.9%)	97/163 (59.5%)	1.01 (0.87–1.17)	
Survival without BPD at 40 weeks’ PMA	PDA ligation	32/55 (58.2%)	23/51 (45.1%)	0.96 (0.64–1.44)	0.42
	No PDA ligation	116/196 (59.2%)	117/205 (57.1%)	1.03 (0.89–1.19)	

^*^Adjusted for gestational age at birth, birthweight, sex, multiple gestation, maternal age, respiratory severity score (RSS) at enrollment.

Among infants who had a PDA ligation (definition B), the incidence of survival without BPD at 36 weeks’ PMA was 27.3% in the late surfactant group compared to 23.5% in the control group (adjusted odds ratio 0.86, 95% confidence interval 0.45–1.66). For infants who did not have a PDA ligation, the incidence of survival without BPD at 36 weeks’ PMA was 32.7% in the late surfactant group compared to 33.8% in the control group (adjusted odds ratio 1.05, 95% confidence interval 0.81–1.36). There was no interaction between a PDA ligation and late surfactant (*p* = 0.84) (Table 3).

Additionally, an interaction between the presence of a PDA and late surfactant did not exist for the outcome of survival without BPD at 40 weeks’ PMA, using PDA definition A (*p* = 0.33) or PDA definition B (*p* = 0.42) (Table 3). An interaction did not exist between the presence of a PDA and receipt of late surfactant for any of the prespecified secondary outcomes using PDA definition A (Table 4). Defining PDA presence as a PDA ligation (definition B) yielded similar results (Table 5).

**Table 4:** Interaction between PDA (definition A) and study secondary outcomes.

Outcome	Subgroups	Late Surfactant *N* = 252	Control *N* = 259	Adjusted odds ratio* (95% CI)	*P* value for interaction
Persistent Pulmonary Morbidity	PDA	27/75 (36.0%)	35/76 (46.1%)	0.84 (0.57–1.25)	0.21
	No PDA	47/133 (35.3%)	44/142 (31.0%)	1.00 (0.72–1.40)	
No Pulmonary Morbidity	PDA	29/75 (29.3%)	16/85 (18.2%)	1.43 (0.82–2.50)	0.43
	No PDA	37/135 (27.4%)	35/144 (24.3%)	1.14 (0.78–1.68)	
BPD at 36 weeks’ PMA	PDA	61/89 (68.5%)	65/94 (69.1%)	0.99 (0.82–1.2)	0.69
	No PDA	93/162 (57.4%)	89/164 (54.3%)	1.06 (0.87–1.28)	
BPD at 40 weeks’ PMA	PDA	33/89 (37.1%)	42/93 (45.2%)	0.82 (0.58–1.17)	0.46
	No PDA	49/162 (30.2%)	51/163 (31.3%)	0.97 (0.7–1.34)	
Death before 36 weeks’ PMA	PDA	3/89 (3.4%)	7/94 (7.4%)	0.45 (0.12–1.7)	0.29
	No PDA	15/162 (9.3%)	15/164 (9.1%)	1.01 (0.51–2)	
Death before 40 weeks’ PMA	PDA	5/89 (5.6%)	8/93 (8.6%)	0.65 (0.22–1.92)	0.44
	No PDA	16/162 (9.9%)	15/163 (9.2%)	1.07 (0.55–2.1)	
Grade 2 or 3 BPD, or death before 36 weeks’ PMA	PDA	31/89 (34.8%)	38/94 (40.4%)	0.86 (0.59–1.25)	0.27
	No PDA	56/162 (34.6%)	50/164 (30.5%)	1.13 (0.83–1.55)	
Grade 3 BPD, or death before 36 weeks’ PMA	PDA	12/89 (13.5%)	12/94 (12.8%)	1.06 (0.5–2.23)	0.71
	No PDA	26/162 (16%)	21/164 (12.8%)	1.25 (0.74–2.13)	
Duration Mechanical Ventilation ≥37 days (Median)	PDA	61/89 (68.5%)	56/95 (58.9%)	1.16 (0.93–1.45)	0.32
	No PDA	70/163 (42.9%)	69/164 (42.1%)	1.02 (0.79–1.31)	
Home Respiratory Support Post-Discharge	PDA	33/74 (44.6%)	47/83 (56.6%)	0.79 (0.57–1.08)	0.69
	No PDA	47/134 (35.1%)	71/140 (50.7%)	0.69 (0.52–0.92)	
Total NICU Days ≥106 days (Median)	PDA	51/89 (57.3%)	51/95 (53.7%)	1.07 (0.82–1.38)	0.67
	No PDA	77/163 (47.2%)	78/164 (47.6%)	0.99 (0.79–1.25)	

^*^Adjusted for gestational age at birth, birthweight, sex, multiple gestation, maternal age, respiratory severity score (RSS) at enrollment.

**Table 5:** Interaction between PDA Ligation (definition B) and study secondary outcomes.

Outcome	Subgroups	Late Surfactant *N* = 252	Control *N* = 259	Adjusted odds ratio* (95% CI)	*P* value for interaction
Persistent Pulmonary Morbidity	PDA ligation	15/48 (31.3%)	20/43 (46.5%)	0.81 (0.50–1.31)	0.24
	No PDA ligation	59/160 (36.9%)	59/175 (33.7%)	1.00 (0.75–1.33)	
No Pulmonary Morbidity	PDA ligation	14/48 (29.2%)	5/49 (10.2%)	2.21 (0.85–5.72)	0.14
	No PDA ligation	45/162 (27.8%)	46/180 (25.6%)	1.14 (0.81–1.60)	
BPD at 36 weeks’ PMA	PDA ligation	40/55 (72.7%)	38/51 (74.5%)	0.98 (0.78–1.23)	0.71
	No PDA ligation	114/196 (58.2%)	116/207 (56%)	1.04 (0.88–1.23)	
BPD at 40 weeks’ PMA	PDA ligation	22/55 (40%)	27/51 (52.9%)	0.76 (0.5–1.14)	0.32
	No PDA ligation	60/196 (30.6%)	66/205 (32.2%)	0.95 (0.71–1.27)	
Death before 36 weeks’ PMA	PDA ligation	0/55 (0%)	1/51 (2%)	NA	0.97
	No PDA ligation	18/196 (9.2%)	21/207 (10.1%)	0.91 (0.5–1.65)	
Death before 40 weeks’ PMA	PDA ligation	1/55 (1.8%)	1/51 (2%)	0.93 (0.06–14.44)	0.99
	No PDA ligation	20/196 (10.2%)	22/205 (10.7%)	0.95 (0.54–1.69)	
Grade 2 or 3 BPD, or death before 36 weeks’ PMA	PDA ligation	18/55 (32.7%)	23/51 (45.1%)	0.73 (0.45–1.18)	0.13
	No PDA ligation	69/196 (35.2%)	65/207 (31.4%)	1.12 (0.85–1.48)	
Grade 3 BPD, or death before 36 weeks’ PMA	PDA ligation	6/55 (10.9%)	3/51 (5.9%)	1.85 (0.49–7.03)	0.50
	No PDA ligation	32/196 (16.3%)	30/207 (14.5%)	1.13 (0.71–1.78)	
Duration Mechanical Ventilation ≥37 days (Median)	PDA ligation	43/55 (78.2%)	34/51 (66.7%)	1.17 (0.92–1.49)	0.26
	No PDA ligation	88/197 (44.7%)	91/208 (43.8%)	1.02 (0.82–1.27)	
Home Respiratory Support Post-Discharge	PDA ligation	22/47 (46.8%)	26/47 (55.3%)	0.85 (0.57–1.26)	0.49
	No PDA ligation	58/161 (36%)	92/176 (52.3%)	0.69 (0.54–0.88)	
Total NICU Days ≥106 days (Median)	PDA ligation	34/55 (61.8%)	33/51 (64.7%)	0.96 (0.71–1.28)	0.68
	No PDA ligation	94/197 (47.7%)	96/208 (46.2%)	1.03 (0.84–1.27)	

^*^Adjusted for gestational age at birth, birthweight, sex, multiple gestation, maternal age, respiratory severity score (RSS) at enrollment.

## Discussion

In this post hoc secondary analysis of extremely preterm infants enrolled in TOLSURF, the presence of a PDA did not modify the effect of late surfactant for any of the evaluated primary and secondary outcomes. On the basis of physiologic rationale and prior data identifying worsening of RSS following receipt of late surfactant, we hypothesized that patients with a PDA may have worse later respiratory outcomes following late surfactant; however, our results do not suggest that PDA presence impacts later outcomes among infants receiving late surfactant.

Our hypothesis was motivated, in part, by findings from Beauchene et al., reporting worsened respiratory severity scores within 48 h of 71 late surfactant doses among a single center cohort of 35 infants born <27 weeks’ gestation [11]. The authors reported an improvement in respiratory severity score following 24/45 (55%) surfactant doses when no PDA was present vs 2/26 doses (8%) when a PDA was present. Additionally, when a PDA was present, a negative change in the respiratory severity score was more common (11/26, 42% with a PDA vs 5/45, 11% without a PDA). These findings raised questions about whether there could be longer-term harm associated with late surfactant administration in infants with a PDA. Our study suggests that the presence or lack of a PDA does not influence later outcomes.

The lack of an interaction between late surfactant and PDA status could have several explanations. First, the changes in respiratory severity score observed by Beauchene et al. may have been transient and not had a meaningful impact on the patient’s later clinical course. Second, as noted, all patients in TOLSURF were receiving iNO at the time of late surfactant or control intervention administration. Physiologically, surfactant administration in the presence of a PDA can lower pulmonary vascular resistance (PVR), increase pulmonary blood flow, and potentially worsen the patient’s respiratory status. However, given the co-administration of iNO, it is possible that any additional reduction in PVR from late surfactant was less significant beyond the iNO effect and attenuated any potential clinical worsening in the PDA group. Finally, there is now a large pool of data showing that the medical and surgical treatments, as currently studied in RCT designs, to induce closure of the patent ductus arteriosus among preterm infants does not improve long term outcomes, such as BPD or death [13–17]. Although there is a strong physiological argument for the harms of a hemodynamically significant PDA shunt (ductal steal causing pulmonary over circulation, increased IVH risk, and increased necrotizing enterocolitis risk), the available data suggests that altering its presence may not reduce these harms. On the contrary, whether the enrolled population in recent trials represents a true disease state is questionable as the adjudication of hemodynamic significance of the PDA was not standardized and was not a comprehensive of shunt volume and limited to evaluation of ductal diameter. Thus, the lack of an interaction observed in this study may be related to limitations with our treatment-based PDA definitions, and results may have been different with an echocardiogram derived hemodynamically significant PDA definition, as performed in the Beauchene et al. study.

Despite the lack of improvement in survival without BPD with late surfactant, there was an improvement in post-discharge respiratory morbidity, offering evidence to support its use. Additionally, surfactant is a good medium for pulmonary drug administration and has been used for the delivery of anti-inflammatory medications such as budesonide. Recent studies have not shown an improvement in long term outcomes for budesonide mixed with surfactant administered in the first 48 h of life [18]; however, there is a lack of evidence with respect to administering budesonide with late surfactant. This could be an area of future investigation, and our study supports the safety of its administration to all infants, including those with a PDA.

This study has limitations. Given lack of echocardiography data, we defined PDA presence by treatment, which can be subjective and may have misclassified the presence of a PDA. In addition, it is not possible to determine whether PDA burden, as determined by shunt severity or duration of exposure, impacts the relationship. As mentioned above, the co-administration of iNO to all infants in the study may have masked any potential worsening of respiratory status in the late surfactant PDA group, if the PVR was already significantly lowered by the iNO. It is possible that our study results may have been different if iNO were not administrated. We did not look at the relationship between severity of BPD or whether there is an impact on pulmonary vascular disease. Finally, all surfactant used in TOLSURF was calfactant, and there is evidence to suggest that the PDAs may dilate or constrict differently when exposed to different surfactants [19]. Thus, it is possible that our results may have been different in the setting of a different surfactant. Finally, site to site variations in respiratory care, PDA management, and other factors can impact the measured outcomes, including BPD. Unfortunately, site level data was not available in the deidentified dataset we had access to for this study. However, given the original study utilized stratified randomization by center, with permuted blocks and two gestational age strata further applied at the site level, we would anticipate that clinical practice variations would be evenly applied to both the intervention and control arms, reducing any impact on this secondary analysis.

This study has several strengths. TOLSURF was a large scale multi-center trial that followed a detailed study protocol with standard definitions for covariates and adjudicated primary outcomes. We used the original study data and outcomes in our design to minimize any bias in our approach.

## Conclusion

The presence of a PDA did not modify the effect of late surfactant among ventilated preterm infants receiving iNO for the outcomes of survival without BPD at 36 or 40 weeks’ PMA, or for all secondary outcomes.

## Data Availability

The datasets analyzed during the current study can be requested from ClinicalTrials.gov (NCT01022580).
